# Glucagon-Like Peptide-1 Receptor Agonists Combined With Personalized Digital Health Care for the Treatment of Metabolic Syndrome in Adults With Obesity: Retrospective Observational Study

**DOI:** 10.2196/63079

**Published:** 2025-03-27

**Authors:** Hala Zakaria, Hadoun Jabri, Sheikha Alshehhi, Milena Caccelli, Joelle Debs, Yousef Said, Joudy Kattan, Noah Almarzooqi, Ali Hashemi, Ihsan Almarzooqi

**Affiliations:** 1 GluCare.Health, Jumeirah 1, Alwasl road, Dubai, UAE, Dubai, 109239, United Arab Emirates, 971 589154424

**Keywords:** metabolic syndrome, obesity, GLP-1 medications, hybrid model of care, digital health, effectiveness, digital engagement, hybrid care, adult, cardiovascular disease, type 2 diabetes, insulin resistance, efficacy, behavioral change, obese, zone health, weight loss, monitoring, tirzepatide, semaglutide, treatment, medication, telehealth, health informatics, glucagon-like peptide-1

## Abstract

**Background:**

Metabolic syndrome (MetS) represents a complex and multifaceted health condition characterized by a clustering of interconnected metabolic abnormalities, including central obesity, insulin resistance, dyslipidemia, and hypertension. Effective management of MetS is crucial for reducing the risk of cardiovascular diseases and type 2 diabetes.

**Objective:**

This study aimed to assess the effectiveness of combining glucagon-like peptide-1 (GLP-1) and dual gastric inhibitory polypeptide (GIP)/GLP-1 agonists with a continuous, digitally delivered behavioral change model by an integrated care team, in treating MetS among individuals with obesity.

**Methods:**

The 6-month Zone.Health (meta[bolic]) weight loss program involved 51 participants (mean age 45, SD 10 years; mean BMI 35, SD 5 kg/m²), categorized by gender, and treated with either tirzepatide or semaglutide. Participants received continuous support via a digital health platform, which facilitated real time monitoring and personalized feedback from an integrated care team. Engagement levels with the digital platform, measured by the frequency of inbound interactions, were tracked and analyzed in relation to health outcomes.

**Results:**

Tirzepatide reduced waist circumference (WC) by −18.08 cm, compared with −13.04 cm with semaglutide (*P*<.001). Triglycerides decreased significantly with both drugs, with tirzepatide showing a reduction of −64.42 mg/dL and semaglutide −70.70 mg/dL (*P*<.001). Tirzepatide generally showed more pronounced improvements in fasting glucose, blood pressure (BP), low-density lipoprotein, and total cholesterol compared with semaglutide. Higher engagement with the digital health platform showed significant difference among the 3 groups; the group with the highest level of app-based interactions (≥25 interactions) had the greatest WC reduction (mean −19.04, SD 7.40 cm) compared with those with ≤15 interactions (mean −9.60, SD 5.10 cm; *P*=.002). Similarly, triglycerides showed the greatest reduction in the group with ≥25 interactions (mean −108.56, SD 77.06 mg/dL) compared with those with ≤15 interactions (mean −44.49, SD 50.85 mg/dL; *P*=.02). This group also exhibited the largest reduction in diastolic BP (mean −10.33, SD 7.40 mm Hg) compared with those with ≤15 interactions (mean −0.83, SD 7.83 mm Hg; *P*=.004), and the most substantial decrease in fasting glucose levels (mean −18.60, SD 10.82 mg/dL) compared with those with ≤15 interactions (mean −2.49, SD 27.54 mg/dL; *P*=.02). Participants in the highest quartile of digital engagement had a 60% greater likelihood of MetS reversal compared with those in the lowest quartile.

**Conclusions:**

This study shows that combining GLP-1 and dual GIP/GLP-1 agonists with a digital behavioral change model significantly improves MetS markers in individuals with obesity. Tirzepatide proved more effective than semaglutide, leading to greater reductions in WC and triglyceride levels, along with better improvements in fasting glucose, BP, and lipid profiles. Higher app-based engagement was linked to better health outcomes, with participants in the highest engagement group having a 60% greater likelihood of treating MetS compared with those with the lowest engagement.

## Introduction

Metabolic syndrome (MetS) represents a complex and multifaceted health condition characterized by a clustering of interconnected metabolic abnormalities, including central obesity, insulin resistance, dyslipidemia, and hypertension [1]. Individuals diagnosed with MetS face an elevated risk of developing cardiovascular disease [2], type 2 diabetes [3], and other related complications [4]. Understanding the intricate mechanisms underlying MetS is crucial for optimizing patient management and outcomes. In the United Arab Emirates, the prevalence of MetS was 33.6% (269/801) in the Emirati population, 34.5% (214/620) in the Arab non-Emirati population, and 40.7% (695/1709) in the Asian non-Arab population [5].

Obesity serves as a primary contributing factor to the development and progression of MetS. Excess adipose tissue, particularly visceral fat, contributes to chronic low-grade inflammation and the release of adipokines, cytokines, and free fatty acids, all of which play vital roles in insulin resistance and metabolic dysregulation [6]. Central obesity, characterized by an accumulation of adipose tissue around the abdomen, is particularly indicative of MetS and poses a heightened risk for cardiovascular complications [7]. The management of MetS revolves around lifestyle modifications and pharmacological interventions aimed at addressing its components and reducing overall cardiovascular risk [8]. Lifestyle interventions, including dietary changes, regular physical activity, and weight management, form the cornerstone of treatment [9]. In patients with MetS, dietary strategies emphasizing a balanced intake of macronutrients and lifestyle modification such as smoking cessation, regular exercise, and proper eating habits may improve profiles of each component of MetS and reduce the risk of developing diabetes and cardiovascular disease [9].

Pharmacotherapy plays a complementary role in the management of MetS, with medications targeting specific components of the syndrome. This may include statins to address dyslipidemia, antihypertensive agents to manage elevated blood pressure (BP), and insulin-sensitizing drugs to improve glucose metabolism [8]. Notably, emerging therapeutic agents such as glucagon-like peptide-1 (GLP-1) receptor agonists have shown promise in addressing multiple aspects of MetS, including glycemic control, weight reduction, and cardiovascular risk reduction [10]. By mimicking the action of endogenous GLP-1, these medications stimulate glucose-dependent insulin secretion, suppress glucagon release, and delay gastric emptying, resulting in improved glycemic control and reduced appetite [10]. Furthermore, GLP-1 receptor agonists have been associated with favorable effects on body weight, BP, and lipid profiles, making them attractive therapeutic options for individuals with MetS [10].

MetS poses a significant challenge to public health, necessitating comprehensive and more engaging approaches for prevention and management. Integrating dietary modifications, lifestyle interventions, and pharmacological treatments targeting specific components of the syndrome are essential for mitigating its adverse outcomes. The Zone.Health’s meta[bolic] program, which combines pharmacotherapy with continuous engagement and monitoring to enable sustainable lifestyle modifications, demonstrated significant improvements in weight, body composition, and metabolic markers [11]. Enhancing traditional treatment paradigms by integrating continuous digital health monitoring with tailored pharmacotherapy. This program leverages advanced analytics and real time data to dynamically adjust treatment plans, fostering deeper patient engagement and more precise management of MetS components. Compared with traditional models that often rely on intermittent follow-ups and generalized treatment approaches, Zone.Health’s continuous care model ensures long-term lifestyle modifications and pharmacological adherence, which are critical for long-term management of MetS. This integration of technology and personalized care is designed to significantly improve clinical outcomes by providing consistent, supportive, and adaptive interventions tailored to individual patient needs. Therefore, the aim of this study is to measure the effectiveness of GLP-1 and GLP-1/gastric inhibitory polypeptide (GIP) medications when combined with a continuous, digitally delivered behavioral change model involving a multidisciplinary team in the treatment of MetS components and weight among individuals with obesity in the UAE population.

## Methods

### Zone.Health Program

Zone.Health, introduced by meta[bolic] [12] in 2023, is a value-based 6-month weight loss program designed to be used in combination with pharmacotherapy. This innovative initiative offers hyperpersonalized insights, and monitoring in order to have patients adopt realistic, bite-sized behavioral change decisions over the duration of the program. The program’s “value-based” approach underscores its commitment to efficacy, promising partial refunds to participants who fulfill specified compliance metrics but do not achieve a minimum 10% weight loss within the program’s timeframe. This ensures accountability while prioritizing the participant’s progress and well-being. Hyperpersonalization lies at the core of Zone.Health, where each participant’s unique physiological, behavioral, and psychological profile is considered. When focusing on physiological factors, parameters like age, fat mass, muscle mass, and physical activity levels are considered. Whereas the behavioral and psychological aspect includes mental health, goal setting, and psychological beliefs. Furthermore, using continuous glucose monitoring (CGM), alongside activity and sleep tracking, and other digital biomarkers, the health care team dynamically tailors dietary recommendations, exercise plans, and medication regimens to suit each participant needs. The ongoing engagement between participants and the health care team is necessary. Biweekly sessions by the health coach comprehensive provides support, addressing progress, challenges, and evolving needs. As participants advance, engagement frequency adjusts to monthly sessions, focusing on maintaining achievements and adapting strategies for long-term success. Zone.Health’s multifaceted approach extends beyond face-to-face interactions, incorporating an intuitive app called “Zone.Health” supported by the health care team including coaches, sports scientists, dietitians, physicians, and diabetes educators. The app is integrated with a nutrition artificial intelligence food logging tool used by participants for food logging as well as integration with wearable data from Apple healthkit or Google fit. Participants are educated by dietitians to ensure correct food logging and minimize errors, by diabetes educators to interpret CGM data, and by health coaches to ensure integrating all other digital wearables (including Apple watch, Google Fitbit, or ŌURA ring [Oura Health Ltd]) into the app and educate the participants data collected from these wearables. Data integration is seamless, with unified and time-synchronized information accessible through a single clinician portal, facilitating effective patient monitoring and engagement. Medication adjustments are made monthly, guided by assessments of fat versus muscle loss ratio, weight fluctuations, and reported side effects. Regular measure of body composition analysis using (Seca mBCA 514, Seca GmbH & Co. KG) ensures a comprehensive understanding of each participant’s progress, with evaluations conducted both at the program’s onset and quarterly thereafter for comparison. The effectiveness of the Zone.Health’s hybrid approach is supported by previous research, particularly in diabetes management, demonstrating its potential to revolutionize weight loss strategies and improve overall health outcomes [11,13,14].

### Study Design

**Figure 1.**
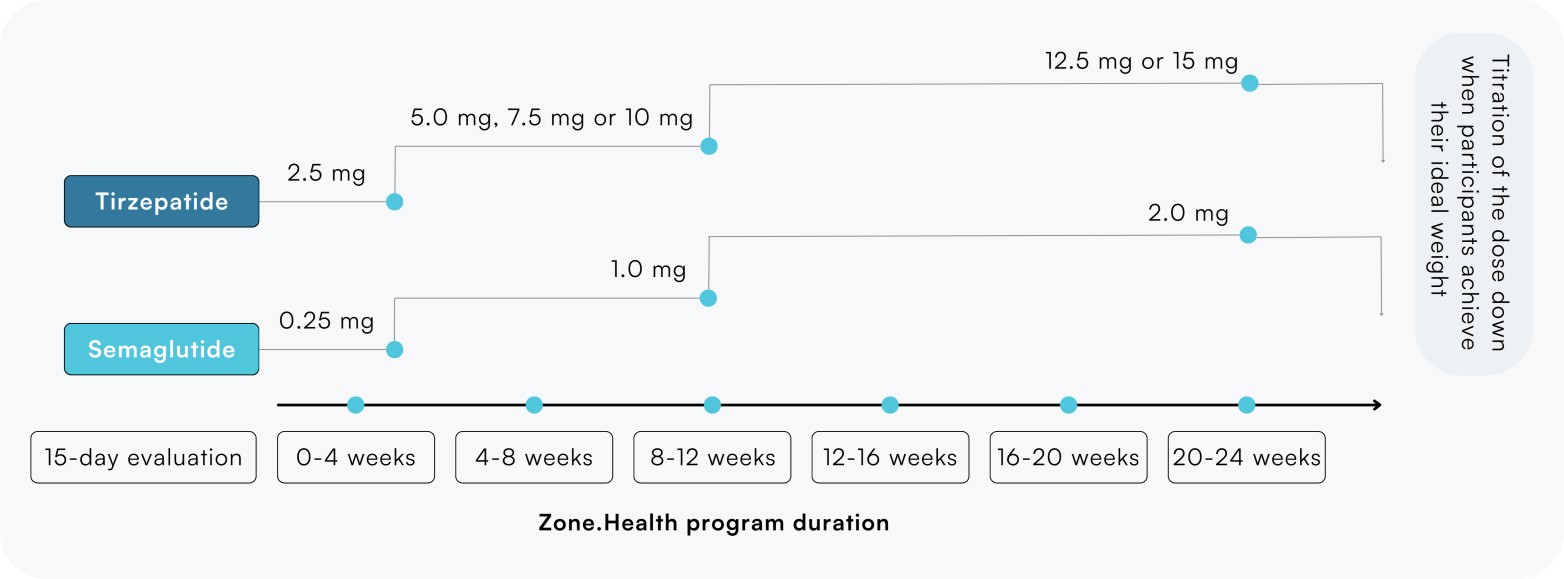
Tirzepatide and semaglutide dose titration throughout the Zone.Health program.

A retrospective, real world evidence observational study was conducted among participants with MetS that signed up to the Zone.Health weight loss program. A comprehensive examination of medical records was undertaken to explore the occurrence and factors contributing to MetS among patients who were prescribed GLP-1 medications. This study encompassed 51 adult patients from diverse ethnic backgrounds who had completed a 6-month program duration with all MetS parameters collected. Medication administration followed a thorough assessment, including a 15-day evaluation period and initial dietary analysis, to confirm participant eligibility. Treatment selection, whether semaglutide or tirezepatide, was determined by physicians based on clinical suitability. Following the standard practice, tirzepatide was started with an initial dose of 2.5 mg, gradually increasing to either 5.0 mg, 7.5 mg, or 10 mg by the 3-month mark and reaching 12.5 mg or 15 mg by 6 months. Semaglutide started at 0.25 mg, escalating to 1 mg at 3 months and reaching to 2.0 mg at 6 months (Figure 1). All participants adhered to the criteria, ensuring the continued validity of contractual agreements. The study strictly followed the ethical guidelines of the Declaration of Helsinki, ensuring each participant gave signed consent. The UAE health care authority monitored the clinical protocols, ensuring they met international ethical standards for research involving human subjects. All study procedures, including data collection and analysis, respected participant rights and privacy.

### Study Outcomes

#### Primary Objective

Reduction in MetS markers (waist circumference [WC], triglycerides, and fasting glucose levels) among individuals with obesity over a 6-month period when treated with GLP-1 (semaglutide) and dual GIP/GLP-1 agonists (tirzepatide).

#### Secondary Objectives

First, changes in BP (systolic and diastolic) and lipid profiles (total cholesterol, low-density lipoprotein [LDL], and high-density lipoprotein [HDL]) in participants receiving tirzepatide versus those receiving semaglutide.

Second, association between levels of digital engagement with the health platform and improvements in MetS markers.

### Criteria for Diagnosing MetS

International Diabetes Federation (IDF) deﬁnition of MetS was used accordingly to classify participants with MetS [15]. In this study, females and males were included in the study if they had the MetS if the following criteria were met.

First, waist circumference of ≥94 cm for males and ≥80 cm for females, based on ethnic-specific values, such as Europids, Sub-Saharan Africans, Ethnic South and Central Americans, and Middle Eastern individuals.

Second, any two of the following four factors: (1) raised triglycerides ≥150 mg/dL (1.7 mmol/L) or specific treatment for this lipid abnormality; (2) reduced HDL cholesterol (males <40 mg/dL [1.03 mmol/L], females <50 mg/dL [1.29 mmol/L]), or specific treatment for this lipid abnormality; raised blood pressure (systolic BP ≥130 mm Hg or diastolic BP ≥85 mm Hg), or treatment for previously diagnosed hypertension; (4) raised fasting plasma glucose ≥100 mg/dL (5.6 mmol/L), or previously diagnosed type 2 diabetes.

### Data Collection and Participants

Data were collected from physicians’ patient records at baseline and at 6 months using the electronic medical record (Diamond, Hicom). Patients’ gender, age, ethnicity, medication, WC (cm), weight kg, BP (mm Hg), and BMI (kg/m²) variables were collected. Laboratory variables were collected including total cholesterol (mg/dL), LDL cholesterol (mg/dL), HDL cholesterol (mg/dL), triglycerides (mg/dL), and fasting glucose (mg/dL). Engagement interactions were collected from the app portal. Inbound interactions refer to interactions or messages received from the participants related to dietary advice or modifications, medication dosage and side effects, coaching-related queries, and inquiries directed at any of the health care team. The outbound interactions refer to the interactions or messages initiated by the health care team to the participants, which consists of (1) biweekly reminders of food logging, weight recording, data integration, and monthly workout schedules (dietitians, coaches, and sport scientists); (2) biweekly physicians follow-up on the side effect of the medications; and (3) CGM data feedback provided by the diabetes educator.

### Participant Inclusion Criteria

Eligible participants were adults (aged 18 years and older) diagnosed with MetS and obesity, who met the IDF criteria for MetS, which includes central obesity (WC specific to population and gender) plus at least 2 of the following: raised triglycerides, reduced HDL cholesterol, raised BP, or raised fasting plasma glucose. Participants had been prescribed GLP-1 or GIP/GLP-1 therapies as part of their treatment regimen. Individuals with incomplete records or those who did not engage with the digital platform were excluded from the analysis.

### Ethical Considerations

Ethical approval was obtained from the Dubai Health Authority (DHA; DSREC-02/2025_09), and patient data were anonymized to maintain confidentiality. To protect participant privacy, all data collected were anonymized, and measures were taken to safeguard sensitive information, including data encryption and restricted access. It is important to note that no compensation was provided to participants for their involvement in this study.

### Statistical Analysis

Data analysis performed using SPSS software version 29.0 (IBM Corp). Continuous data (such as age, weight, and laboratory parameters) were presented as means and SDs, and categorical variables (such as gender, medication type, Total n° of IDF criteria met, and ethnicity) were expressed as counts and percentage. The paired *t* test was used to compare variables at baseline and at 6 months and ANOVA test was used to see the baseline characteristics between female and male. One-way ANOVA test was conducted to test differences in baseline characteristics across medication types. ANOVA was also used to assess differences between app-based interaction groups and their impact on improvements in MetS markers. *P*<.05 was considered significant. Furthermore, post hoc Bonferroni test was conducted to measure the difference between app-based engagement groups.

## Results

### Basic Demographics and Baseline Characteristics Stratified by Sex (N=51)

The study population is almost evenly divided between individuals of Middle Eastern (23/51, 45%) and European (28/51, 55%) ethnicities (Table 1 and Table 2). Majority of participants are treated with tirzepatide (31/51, 61%) as opposed to semaglutide (20/51, 39%). In terms of the number of MetS parameters met, all patients met the IDF criteria with most participants (34/51, 67%) meeting 3 criteria, 27% (14/51) meeting 4, and a small group of 6% (3/51) meeting all 5 criteria. Differences between the tirzepatide and semaglutide groups are evident in several baseline characteristics. There is a significant difference in age, with the tirzepatide group being older (*P*=.02). Notable disparities also exist in weight and WC, with the tirzepatide group being significantly heavier (*P*=.01) and having a larger WC (*P*=.002). The tirzepatide group also has a higher BMI (*P*=.02). BP measurements between the groups are similar, without significant differences in either systolic or diastolic BP. Cholesterol profiles are comparable, with no significant differences in total cholesterol, LDL cholesterol, or HDL cholesterol levels. In addition, there are no significant differences in triglycerides or fasting glucose levels between the 2 groups.

**Table 1.** Basic demographics and baseline characteristics in all participants classiﬁed as having the metabolic syndrome according to the IDF^a^ criteria.

Characteristic		Values (N=51), n (%)
Ethnicity		
	Middle Eastern	23 (45)
	European	28 (55)
Sex		
	Female	28 (55)
	Male	23 (45)
Total number of IDF criteria met		
	3	34 (67)
	4	14 (27)
	5	3 (6)

a IDF: International Diabetes Federation.

**Table 2.** Clinical and metabolic characteristics of participants on tirzepatide and semaglutide.

Characteristics	Tirzepatide (n=31), mean (SD)	Semaglutide (n=20), mean (SD)	*P* value
Age (years)	47.03 (10.49)	40.05 (9.61)	.02^a^
Weight (kg)	105.90 (26.60)	88.61 (14.23)	.01^a^
BMI (kg/m^2^)	35.43 (6.90)	31.49 (3.04)	.02^a^
Systolic BP^b^ (mm Hg)	127.52 (14.66)	123.55 (13.00)	.33
Diastolic BP (mm Hg)	79.03 (8.90)	76.80 (7.82)	.36
Total cholesterol (mg/dL)	198.11 (46.69)	204.52 (40.03)	.62
LDL^c^ cholesterol (mg/dL)	138.39 (43.91)	149.25 (45.42)	.40
Waist circumference (cm)	114.26 (17.64)	100.33 (9.72)	.002^a^
HDL^d^ cholesterol (mg/dL)	47.51 (10.61)	50.93 (11.40)	.28
Triglycerides (mg/dL)	156.41 (70.21)	171.41 (83.93)	.50
Fasting glucose (mg/dL)	106.02 (10.16)	101.72 (22.43)	.38

a *P* values <.05 were considered significant from 1-way ANOVA test.

b BP: blood pressure.

c LDL: low-density lipoprotein.

d HDL: high-density lipoprotein.

### Improvements of Diagnostic Criteria of MetS After 6 Months of Zone.Health Program

Table 3 illustrates significant improvements in the criteria used to diagnose MetS after a 6-month program, comparing the effects of tirzepatide and semaglutide (N=51). Tirzepatide showed greater reductions in WC (–18.08 cm) compared with semaglutide (−13.04 cm), both statistically significant (*P*<.001). Triglyceride levels decreased significantly with tirzepatide (−64.42 mg/dL) and even more with semaglutide (−70.70 mg/dL; *P*<.001). HDL cholesterol increases were modest and not statistically significant for both drugs. Significant reductions were observed in fasting glucose, systolic and diastolic BP, and LDL cholesterol, with tirzepatide generally showing more pronounced improvements than semaglutide. Overall cholesterol levels also decreased significantly in both treatment groups.

**Table 3.** Improvements of diagnostic criteria of metabolic syndrome after 6 months of the program in both female and male (N=51).

Variables	Tirzepatide (n=31)			Semaglutide (n=20)		
	Mean (SD)	*t* test (df)	*P* value	Mean (SD)	*t* test (df)	*P* value
Weight (kg)	−14.07 (5.83)	−13.44 (30)	<.001^a^	−13.38 (4.69)	−12.75 (19)	<.001^a^
BMI (kg/m^2^)	−4.96 (2.54)	−10.69 (29)	<.001^a^	−4.53 (1.89)	−10.69 (19)	<.001^a^
Waist circumference (cm)	−18.08 (8.30)	−12.13 (30)	<.001^a^	−13.04 (7.27)	−8.02 (19)	<.001^a^
Triglycerides (mg/dL)	−64.42 (53.10)	−6.44 (29)	<.001^a^	−70.70 (69.10)	−4.58 (19)	<.001^a^
HDL^b^ cholesterol (mg/dL)	2.50 (8.47)	1.68 (30)	.10	1.97 (8.67)	1.01 (19)	.32
Fasting glucose (mg/dL)	−15.32 (11.46)	−7.07 (27)	<.001^a^	−10.25 (23.64)	−1.89 (18)	.07
Systolic BP^c^ (mm Hg)	−14.74 (13.92)	−5.90 (30)	<.001^a^	−14.75 (10.76)	−6.12 (19)	<.001^a^
Diastolic BP (mm Hg)	−7.23 (9.20)	−4.37 (30)	<.001^a^	−6.00 (8.32)	−3.22 (19)	.004
LDL^d^ cholesterol (mg/dL)	−24.46 (32.58)	−4.04 (28)	<.001^a^	−15.15 (23.64)	−1.86 (18)	.08
Cholesterol (mg/dL)	−28.87 (45.02)	−3.51 (29)	.001^a^	−20.09 (39.98)	−2.13 (17)	.05^a^

a *P* values <.05 were considered significant from paired sample *t* test.

b HDL: high density lipoprotein.

c BP: blood pressure.

d LDL: low-density lipoprotein.

### Treatment of Remaining MetS Markers in Patients With Increased WC (n=26)

In participants who did not reverse MetS due to persistently increased WC, improvements were observed with the remaining MetS markers. For instance, 85% (22/26) of participants successfully treated their elevated triglyceride levels to within normal ranges. HDL saw a significant improvement with 73% (19/26) of participants elevating their levels to the desired range. In addition, BP was stabilized in 89% (23/26) of these participants, and blood sugar levels were brought back to healthy levels in 96% (25/26) of those who did not treat MetS due to persistent increased WC.

### Change in MetS Risk Factors by App-Based Interaction Groups

Table 4 and Table 5 presents the difference between patient engagement and improvements in MetS markers over a 6-month period among all participants. Engagement is quantified as both inbound and outbound interactions; inbound interactions (received from participants) average at 28.86 (SD 21.32), outbound (sent to participants from the health care team) at 58.57 (SD 25.26), and the total number of interactions at 84.00 (SD 45.76). Significant improvements were observed in several markers. WC, triglycerides, diastolic BP, and fasting glucose showed significant changes, with *P* values of .003, .01, .003, and .03, respectively. Changes in HDL and systolic BP were profound but not statistically significant. The post hoc analysis showed significant differences among interaction groups, indicating that higher app-based interactions are associated with better outcomes (Table 6). A significantly increased reduction in WC was found in individuals with ≥25 interactions (mean −19.04, SD 7.40) compared with individuals with ≤15 interactions (mean −9.60, SD 5.10 cm, *P*=.002). In addition, individuals with ≥25 interactions showed a significant reduction in triglycerides (mean −108.56, SD 77.06 mg/dL) compared with those with ≤15 interactions (mean −44.49, SD 50.85 mg/dL, *P*=.02) and a significant reduction when compared with those with 16‐24 interactions (mean −55.77, SD 44.52 mg/dL, *P*=.03). Diastolic BP also significantly decreased in individuals with ≥25 interactions (mean −10.33, SD 7.40 mm Hg) compared with those with ≤15 interactions (mean −0.83, SD 7.83 mm Hg, *P*=.004). Fasting glucose levels were significantly lower in individuals with ≥25 interactions (mean −18.60, SD 10.82 mg/dL) compared with those with ≤15 interactions (mean −2.49, SD 27.54 mg/dL, *P*=.02).

**Table 4.** Distribution of engagement interactions by category.

Engagement interactions and category	Mean (SD)
Inbound	28.86 (21.32)
Outbound	58.57 (25.26)
Total	84.00 (45.76)

**Table 5.** Change in metabolic syndrome risk factors by app-based interaction groups (N=51).

Improvements in MetS^a^ markers	Inbound app-based interactions			
	≤15 (n=12)	16-24 (n=12)	≥25 (n=27)	*P* value
WC^b^ (cm)	−9.60 (5.10)	−16.01 (9.20)	−19.04 (7.40)	.003^c^
Triglycerides (mg/dL)	−44.49 (50.85)	−55.77 (44.52)	−108.56 (77.06)	.01^c^
HDL^d^ (mg/dL)	+1.69 (10.03)	+2.93 (7.33)	+3.15 (5.63)	.85
Systolic BP^e^ (mm Hg)	−12.58 (10.39)	−14.33 (12.79)	−15.89 (13.78)	.76
Diastolic BP (mm Hg)	−0.83 (7.83)	−4.58 (9.29)	−10.33 (7.40)	.003^c^
FG^f^ (mg/dL)	−2.49 (27.54)	−13.41 (8.88)	−18.60 (10.82)	.03^c^

a MetS: metabolic syndrome.

b WC: waist circumference.

c *P* values <.05 were considered significant from 1-way ANOVA test.

d HDL: high-density lipoprotein.

e BP: blood pressure.

f FG: fasting glucose.

**Table 6.** Post hoc analysis of metabolic syndrome markers by interaction group (N=51).

Metabolic syndrome markers and interaction group (I)		Interaction group (J)	Mean difference (I–J)	*P* value
WC^a^ (cm)				
	≤15	16‐24	6.41	.12
	≤15	≥25	9.44^b^	.002^b^
	16‐24	≤15	−6.42	.12
	16‐24	≥25	3.03	.73
	≥25	≤15	−9.44^b^	.002^b^
	≥25	16‐24	−3.03	.73
Triglycerides (mg/dL)				
	≤15	16‐24	11.28	≥.99
	≤15	≥25	64.07^b^	.02^b^
	16‐24	≤15	−11.28	≥.99
	16‐24	≥25	52.79^b^	.03^b^
	≥25	≤15	64.07^b^	.02^b^
	≥25	16‐24	−52.79^b^	.03^b^
Diastolic BP^c^ (mm Hg)				
	≤15	16‐24	3.75	.76
	≤15	≥25	9.50^b^	.004^b^
	16‐24	≤15	−3.75	.76
	16‐24	≥25	5.75	.13
	≥25	≤15	−9.50^b^	.004^b^
	≥25	16‐24	−5.75	.13
FG^d^ (mg/dL)				
	≤15	16‐24	10.92	.35
	≤15	≥25	16.10^b^	.02^b^
	16‐24	≤15	−10.92	.35
	16‐24	≥25	5.19	≥.99
	≥25	≤15	−5.19	≥.99
	≥25	16‐24	−16.10^b^	.02^b^

a WC: waist circumference.

b Mean difference is considered significant at .05 from Bonferroni post hoc test.

c BP: blood pressure.

d FG: fasting glucose.

## Discussion

### Principal Findings

This study examines the effect of GLP-1 and dual GIP/GLP-1 agonists when combined and integrated with a tech-enhanced, continuous feedback cycle on lifestyle modifications in treating MetS. MetS is intricately linked to central obesity and excessive weight, with studies illustrating that increased adiposity, particularly in the abdominal region, is a major risk factor for developing MetS [16]. Central obesity is associated with increased insulin resistance, which plays a pivotal role in the pathogenesis of MetS [16].

Studies have shown that even a modest amount of weight loss is associated with improvement in MetS parameters and related cardiovascular disease risk components. Losing weight is associated with a decrease in insulin resistance and insulin level, decreasing the risk of type 2 diabetes or improving previously diagnosed diabetes [17]. Losing weight also causes a positive impact on dyslipidemia by an increase in the HDL level and a drop in the LDL level [18,19]. GLP-1 and GIP agonist use has been shown to reduce MetS severity, abdominal obesity, and inflammation [20].

Over a 6-month period, the comparative analysis of tirzepatide and semaglutide in the Zone.Health program showed significant improvements in MetS markers. Tirzepatide led to greater reductions in WC and LDL cholesterol, with slightly better results in fasting glucose and BP than semaglutide. While both treatments modestly increased HDL cholesterol without significance, semaglutide had a slightly better effect on lowering triglycerides. This suggests the treatment choice may vary based on individual patient needs and goals.

Despite significant improvements in WC observed in the study, it is noted that the rate of change might be perceived as low relative to other MetS markers. This could be attributed to several factors. First, central obesity, characterized by high WC, is often more stubborn and resistant to reduction through conventional weight loss measures alone [21]. This resistance could be partly due to the physiological characteristics of adipose tissue in the abdominal area, which is more metabolically active and sensitive to hormonal influences [21]. This may be attributed to the physiological characteristics of abdominal adipose tissue, which is highly metabolically active and hormonally sensitive, making it particularly challenging to reduce through pharmacotherapy and lifestyle changes alone. In addition, the IDF criteria consider an increased WC as a mandatory component for the diagnosis of MetS, which could preserve the perceived low reversal rate in study participants despite other significant improvements. The other MetS markers such as triglycerides, fasting glucose, and BP showed more substantial improvements even with persistent increased WC. These markers often respond more dynamically to metabolic improvements induced by GLP-1 agonists and lifestyle interventions. GLP-1 agonists improve insulin sensitivity and secretion, which directly impacts glucose control and lipid metabolism [18,19]. This improvement in insulin dynamics could explain the significant decrease in triglycerides and fasting glucose [22]. Similarly, the improvements in BP may be due to the weight loss itself as well as the improvement in arterial stiffness and endothelial function resulting from better metabolic control [23]. Furthermore, the baseline WC of our participants was significantly higher than the general thresholds for MetS, suggesting that longer-term or more intensive treatment strategies might be necessary to achieve meaningful reductions in WC. Challenges in reducing WC in this study align with findings from the Look AHEAD trial, where intensive lifestyle interventions resulted in significant weight loss but less pronounced reductions in WC [24]. This discrepancy highlights the complexity of central obesity as a metabolic entity and underscores the need for targeted strategies that specifically address this component of MetS.

### Integrating Digital Solutions in Weight Loss Management and MetS Markers

The integration of hybrid care models in chronic disease management, such as diabetes mellitus, has shown promising results in reducing key metrics like HbA1c levels [11,13,14]. Our study also explores the perspective of people with MetS receiving treatment with medication such as semaglutide or tirzepatide along with feedback relating to their health in a real world environment. The long-term treatment of obesity, diabetes, and other chronic diseases requires that health care professionals shift away from episodic care and keep patients continuously engaged. The integration of digital tools as part of a standard care pathway allows health care practices to monitor and communicate with patients by observing personal data received from patients, whether it be activity, sleep habits, or information on dietary choices. Together, they are collectively able to take action regarding medication titration or a more consolidated plan during treatment [25]. The association between patient engagement and improvements in MetS markers, as presented in Table 3 of our study, further underscores the potential of digital health solutions in chronic disease management, particularly when combined with pharmacotherapy. Our findings indicate that higher engagement in inbound interactions is linked to better outcomes in key MetS parameters such as WC, triglycerides, and diastolic BP, and are supported by previous studies around engagement and outcomes in diabetes [13]. The significant reduction in these MetS markers with increased digital interaction suggests that patient engagement may be as critical as medication adherence in the management of MetS. This aligns with the broader trend in health care that emphasizes patient-centered models where ongoing patient engagement and personalized interaction play pivotal roles. The integration of digital tools facilitates this by providing continuous monitoring and real time feedback, which are essential for sustaining behavior changes over time. Furthermore, the Peterson Report emphasizes that purely digital solutions often fall short in achieving long-term patient engagement and effective disease management, citing a lack of personalization and direct human interaction as key shortcomings [26]. This finding supports the adoption of a hybrid care model, which combines the scalability of digital tools with the tailored support and expertise of the treating health care professionals.

To our knowledge, this study is the first to examine the effectiveness of GLP-1 medications alongside lifestyle interventions under a hybrid model of care in improving MetS markers. However, this study has a few limitations. First, the sample size, although adequate for detecting significant changes, is not large enough to ensure the generalizability of the findings across diverse populations or to detect more subtle effects. Second, the absence of a control group makes it difficult to definitively attribute the improvements in MetS markers to the interventions, as comparisons with a nonintervention baseline are lacking. In addition, Zone.Health is a paid program, and monetary investments into the program may have influenced participant outcomes, as those who have paid for the program might be more likely to participate actively and follow instructions. What can be concluded is that GLP outcomes on certain MetS parameters can be enhanced when delivered in a hybrid care model, as demonstrated in previous studies, thereby leading to better outcomes, an important factor considering the cost of GLP treatments.

### Conclusions

In conclusion, this study highlights the effective role of combining GLP-1 and GIP/GLP-1 agonists with a continuous, digitally delivered behavioral change model by an integrated care team in managing MetS, particularly in reducing WC, improving lipid profiles, and enhancing glycemic control across different genders. Despite notable improvements in most MetS markers, the persistence of increased WC suggests a need for strategies specifically targeted at abdominal obesity. Future research should focus on optimizing interventions that specifically address this resistant aspect of MetS, potentially incorporating new digital biomarker monitoring to enhance engagement even further due to the relationship between engagement and outcomes, as shown in this study.

## Abbreviations

BP: blood pressure
CGM: continuous glucose monitoring
GIP: gastric inhibitory polypeptide
GLP-1: glucagon-like peptide-1
HDL: high-density lipoprotein
IDF: International Diabetes Federation
LDL: low-density lipoprotein
MetS: metabolic syndrome
WC: waist circumference
